# Polyphosphates and Complement Activation

**DOI:** 10.3389/fmed.2019.00067

**Published:** 2019-04-04

**Authors:** Edward M. Conway

**Affiliations:** Division of Hematology, Department of Medicine, Faculty of Medicine, Centre for Blood Research, Life Sciences Institute, University of British Columbia, Vancouver, BC, Canada

**Keywords:** innate immunity, thrombosis, inflammation, coagulation, age-related macular degeneration, mouse models, C1-esterase inhibitor, membrane attack complex

## Abstract

To sustain life in environments that are fraught with risks of life-threatening injury, organisms have developed innate protective strategies such that the response to wounds is rapid and localized, with the simultaneous recruitment of molecular, biochemical, and cellular pathways that limit bleeding and eliminate pathogens and damaged host cells, while promoting effective healing. These pathways are both coordinated and tightly regulated, as their over- or under-activation may lead to inadequate healing, disease, and/or demise of the host. Recent advances in our understanding of coagulation and complement, a key component of innate immunity, have revealed an intriguing linkage of the two systems. Cell-secreted polyphosphate promotes coagulation, while dampening complement activation, discoveries that are providing insights into disease mechanisms and suggesting novel therapeutic strategies.

## Introduction

Two major blood-borne proteolytic cascades, complement and coagulation, are fully integrated to cooperatively fight infections and prevent excessive bleeding from wounds. Interplay between these systems is evolutionarily conserved, as evident in the horseshoe crab, a “living fossil” representing arthropods from 500 million years ago (1–3). The released contents of hemocytes induce clotting and destroy invading pathogens and toxins. Complement and coagulation pathways in mammals now appear more distinct, but interactions are increasingly being recognized. Elucidation of the links is yielding novel treatments, including, eculizumab, an effective anti-complement antibody that prevents thrombosis in paroxysmal nocturnal hemoglobinuria (PNH) and atypical hemolytic uremic syndrome (aHUS) (4, 5). More therapies for other common diseases will undoubtedly enter the clinic in coming years (6).

In this report, I review the complement system, highlighting some key mechanisms by which it is regulated, and how it interfaces with coagulation [Readers are referred to excellent reviews of the coagulation cascade (7–10)]. I then focus on recently uncovered insights into the role of the polyanion polyphosphate (polyP)—known to promote coagulation—in dampening complement activation. This will be followed by a discussion of how such an apparent dichotomy in the function of polyP is physiologically relevant.

## Complement Activation

**Figure 1:**
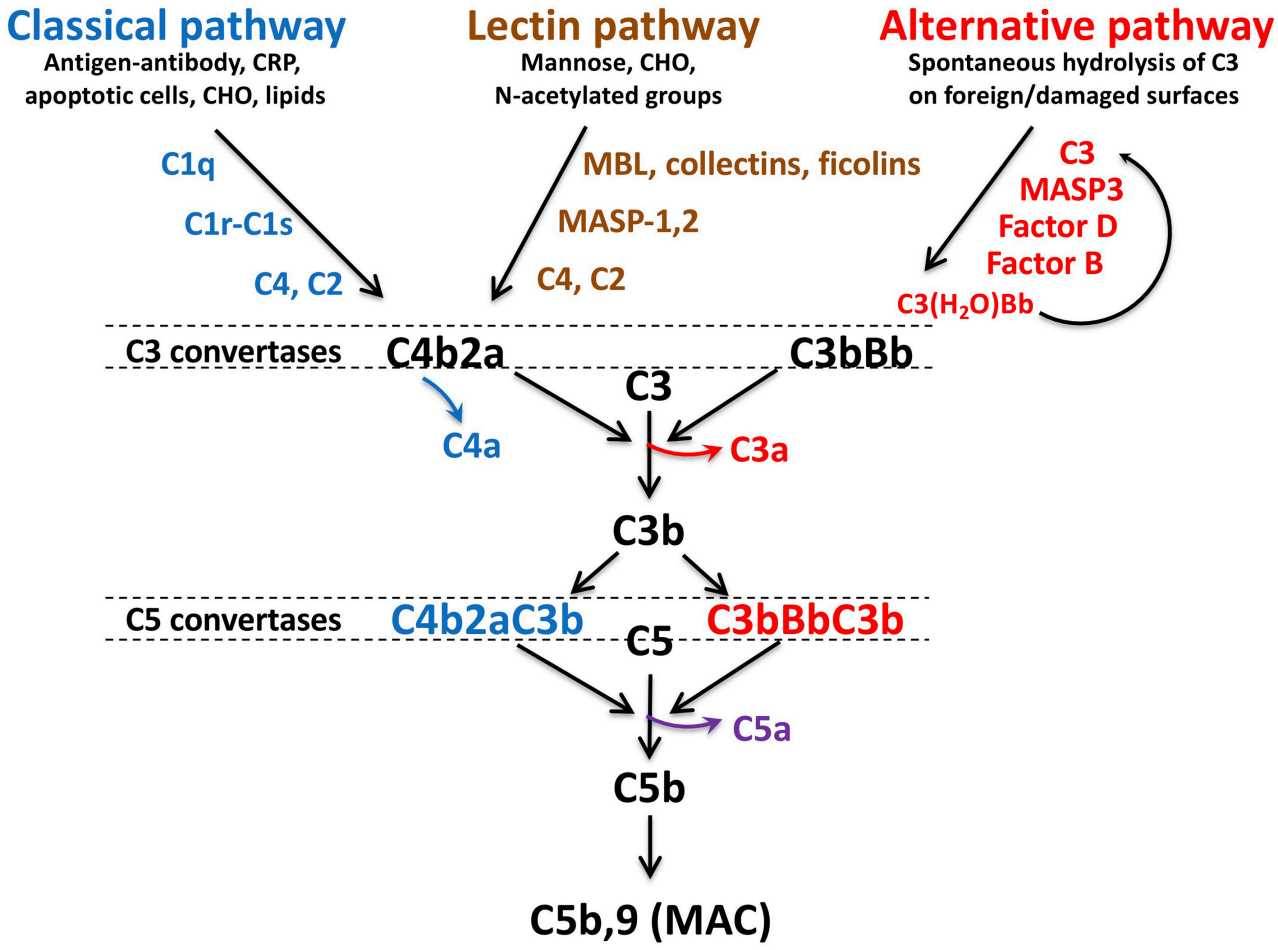
Schematic of complement activation pathways. Complement activation proceeds via the classical, lectin, or alternative pathways, triggered by exposure of surveillance molecules, C1q, MBL, collectins and ficolins, of specific danger signals. The alternative pathway is constitutively “on,” due to spontaneous hydrolysis of C3. The pathways converge to form C3 convertases: C4b2a for the classical and lectin pathways, C3bBb for the alternative pathway. C4a and C3a, are released with cleavage of C4 and C3, respectively. As C3b is further generated, C5 convertases C4bBbC3b and C3bBbC3b are formed, resulting in liberation of the most potent anaphylatoxin C5a, in conjunction with C5b. C5b is the initial component required for spontaneous assembly of the C5b-9 membrane attack complex (MAC) which polymerizes and induces lysis of the cellular target. MBL, mannose binding lectin, CRP, C-reactive protein, CHO, carbohydrate, MASP, MBL associated serine protease. Figure and legend from Conway (14).

Comprising over 30 soluble and membrane-bound proteins, complement contributes to innate immunity and provides a bridge to adaptive immunity (11–13). Complement activation is triggered by exposure of blood to damage-associated molecular patterns that include, for example, pathogens, host DNA from damaged cells, lipids and oligosaccharides (Figure 1). Three pathways—lectin (LP), classical (CP), and alternative (AP)—converge with generation of C3 convertases that proteolyse C3 into C3b and release the anaphylatoxin C3a. The CP is triggered by C1q recognition of antibodies bound to antigens or microbial surfaces. C1q may also recognize other targets, such as C-reactive protein, apoptotic cells, and microbes. It circulates in complex with zymogens of serine proteases C1r and C1s (C1qr^2^s^2^). When C1q binds to its target, C1r autoactivates and activates C1s (15) which in turn cleaves C4, releasing C4a, and C4b, the latter which covalently binds to target surfaces. C2 binds to immobilized C4b and is cleaved by C1s into C2b and C2a, allowing C2a to complex with C4b and form the CP C3 convertase, C4b2a. The LP is similar to the CP (16), but pathogen recognition comprises mannose binding lectin (MBL), ficolins and/or collectin-11. These circulate bound to MBL-associated zymogens of serine proteases MASP1/MASP3 and MASP2 and bind to sugars or N-acetylated groups on micro-organisms. MASP1 autoactivates and cleaves C2 and activates MASP2, while MASP2 cleaves C2 and C4, yielding the C4b2a LP C3 convertase (17).

The AP is constitutively active, sustained by a “tick-over” mechanism in which small amounts of C3 are hydrolyzed to C3(H_2_O) (18), exposing a binding site for factor B (FB). Circulating factor D (FD) cleaves FB into Ba and Bb, the latter which binds to C3(H_2_O) to form a fluid-phase C3 convertase which cleaves C3 to C3b and C3a. With exposure to a pathogen or damaged cell, more C3a and C3b are generated (19), resulting in formation of the AP C3 convertase, C3bBb. Complement is amplified via the AP as the pathways converge and form cell bound C3 convertases. The additional C3b binds to C4b2b and C3bBb, yielding C4bBb(C3b)_n_ and C3bBb(C3b)_n_, which thus become C5 convertases, cleaving C5 into C5b and C5a. C5b is the trigger for the terminal pathway, which spontaneously proceeds with sequential assembly of C6, C7, C8, and multiple C9 subunits, forming the C5b-9 pore-like, lytic membrane attack complex (MAC) that targets invading pathogens and promotes prothrombinase assembly and tissue factor (TF) activation (20).

C5a is a pleiotropic biologically active peptide, exhibiting potent anaphylatoxin properties. C5a also triggers coagulation and inflammation via TF by endothelial cells and monocytes, release/exposure of VWF and P-selectin by endothelial cells and platelets, secretion of inflammatory cytokines, expression of leukocyte adhesion molecules, and release of platelet granule contents that further promote coagulation and complement activation (21–24).

## Regulation of Complement

Complement activation is down-regulated at numerous steps. This ensures a highly localized and temporally appropriate response that spares the host from undesired damage. Acquired or genetic alterations in factors that regulate complement are commonly associated with disease, often featuring varying degrees of vascular-thrombosis. Characterization of these pathways is revealing novel strategies for drug development (6, 25, 26). In the following, I describe a few of the mechanisms by which complement activation is dampened. References for more comprehensive reviews are provided (13, 25, 27–29).

C1-esterase inhibitor (C1-INH) is a serine protease inhibitor that highlights the coordinated regulation of coagulation and complement (10). In coagulation, C1-INH interferes with the proteolytic activities of factor XIa, factor XIIa, and kallikrein, suppressing the contact/intrinsic pathways of coagulation and inflammation. In complement, C1-INH interferes with C1r, C1s, MASP1, and MASP2, preventing formation of the CP/LP convertases. The inhibitory activity of C1-INH is variably potentiated by polyanions, such as heparin (15, 30). Thus, heparin augments C1-INH inhibition of factor XIa and MASP2 (31), but actually dampens C1-INH neutralization of factor XIIa (32), and has almost no effect on C1-INH inhibition of kallikrein, C1r or MASP1 (32, 33). These differential effects of C1-INH that are partly dependent on the cofactor activity of the polyanion, heparin, may help explain why functional deficiencies of C1-INH are not associated clinically with thrombosis.

The major fluid-phase negative regulator of the AP is factor H (FH) (34). Synthesized primarily by the liver, but also by endothelial cells and platelets, FH binds to C3b and glycosaminoglycans of host cells where it suppresses complement by acting as a cofactor for protease factor I (FI) mediated inactivation of C3b to iC3b, accelerating decay of the AP C3 convertase, and competing with FB binding to C3b. Patients with FH mutations are at increased risk of developing aHUS and cardiovascular disease (35–37). FH also binds to VWF in Weibel-Palade bodies and/or facilitates ADAMTS13-mediated proteolysis of ultra large VWF (38–41), providing protection against thrombosis.

The membrane-bound complement receptor (CR)1, glycosylphosphatidylinositol (GPI)-linked CD55, and CD46 are also decay accelerating factors for C3b-containing convertases. Moreover, CR1 and CD46 promote FI-mediated proteolysis of C3b to iC3b (42). Interesting for their distinct clinical presentations, CD46 deficiency is implicated in aHUS (43), while CD55 deficiency is associated with PNH (44).

Assembly and function of the MAC are also regulated to limit host cell damage. The GPI-linked CD59, deficiency of which is also associated with PNH (45), binds to C8 and C9 and prevents C9 polymerization (46). Clusterin binds to C7, C8, and C9, inducing structural changes that reduce integration of C5b-9 into the membrane (47). Vitronectin also prevents C5b-9 membrane binding by promoting formation of a soluble C5b-7 complex (48).

The activities of C3a and C5a are reduced via several coagulation-related enzymes. C5a is proteolysed by plasmin and matrix metalloproteinase 12 (49), while carboxypeptidase B2 (CPB2) [also referred to as activated thrombin-activatable fibrinolysis inhibitor (TAFIa)] (50), reduces the activity of C3a and C5a by cleaving their C-terminal basic amino acids.

### PolyP, Coagulation, and Complement

PolyP is a ubiquitously expressed, linear, anionic polymer of monophosphate units, linked by phosphoanhydride bonds (51). Polymer lengths vary from ~25 to 1,000 units in mammalian cells (52), extending to thousands of units in some bacteria (53). It is abundant in the dense granules of platelets (54, 55), released to the cell surface and/or into the circulation upon activation (55, 56), likely in a charge-neutral form, bound to divalent cations (Ca^2+^, Mg^2+^) and amines (54). Rather than acting as a calcium ion chelator and anticoagulant, polyP promotes coagulation at multiple steps in the cascade (57–61). Long chain polyP is believed to provide a template for autoactivation of factor XII, thereby also triggering inflammation via factor XIIa-mediated activation of the kallikrein-kinin system (62). Shorter forms of polyP released particularly from platelets, bind directly to thrombin (63), fibrinogen, (64), factors XI and XII, pre/kallikrein, high molecular weight kininogen and VWF (56), amplifying generation of factor XIa and thrombin, enhancing the activation of factor V, inactivating tissue factor pathway inhibitor (TFPI), and integrating into the fibrin clot, rendering it more resistant to fibrinolysis (57). The physiologic relevance of several of these and other polyP-coagulation/fibrinolysis protein interactions remain incompletely understood, but targeting polyP is gaining wide interest as a safe anti-thrombotic (65).

**Figure 2:**
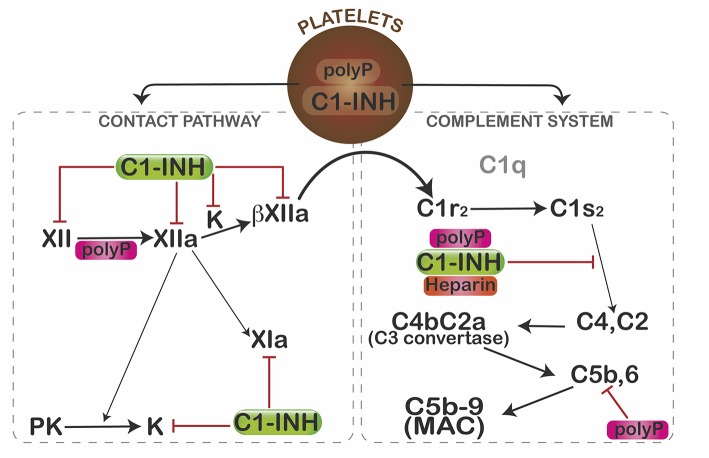
Mechanisms by which polyP regulates complement activation. In resting platelets, polyP and C1-INH are housed in different organelles. After activation, polyP and C1-INH coalesce toward the center of the platelets where they colocalize and are subsequently secreted (66). PolyP triggers a conformational change in factor XII, resulting in generation of XIIa, which can activate prekallikrein (PK) and/or factor XI to XIa. Kallikrein (K) or plasmin (not shown) can further cleave XIIa to generate βXIIa which may activate C1r and thus promote complement activation. C1-INH dampens that pathway by inhibiting factor XII, XIIa, βXIIa, and kallikrein (K). C1s cleaves C4 and C2 to generate the C4b2a C3 convertase, which ultimately leads to formation of the C5b,6 complex, and assembly of the C5b-9 membrane attack complex (MAC). PolyP or heparin potentiate the inhibitory function of C1-INH via direct interactions with C1-INH and the target protease, C1s. PolyP also destabilizes C5b,6, thereby dampening formation of the MAC. Interestingly, in spite of binding to factors XIa and XIIa, reduced levels or function of C1-INH do not cause thrombosis, possibly due in part to differential effects of polyanions (polyP, heparin) on the function of the target enzymes. The over-riding effect of polyP in a serum-based endothelial cell culture system is to suppress complement activation. Figure and legend from Wijeyewickrema et al. (66).

In view of its profound pro-coagulant effects, we predicted that polyP would similarly activate complement. Somewhat surprising, polyP did exactly the opposite (Figure 2). In a C1-INH-dependent manner, polyP dampened C1s-mediated cleavage of C4 and C2 in gel-based assays and cell systems (67). Binding studies revealed that C1-INH directly interacts with the serine protease domain of C1s at a rate that is augmented ~90-fold by the presence of polyP—an effect similar to that seen with heparin. Not formally tested, the data suggested that polyP similarly potentiates C1-INH interactions with MASP2. Interestingly, like heparin, polyP had little potentiating effect on C1r (33). However, these parallels with heparin are limited, since heparin accelerates neutralization of thrombin by antithrombin (AT) >2,000-fold (68), whereas polyP has no effect on the thrombin-AT interaction (57).

PolyP also significantly interferes with activation of the terminal pathway of complement (69) in a size and concentration-dependent manner. It destabilizes C5b-6, reducing the ability of C5b-7 and C5b-8 to bind to and integrate into the target membrane. Other pathways by which polyP might modulate complement activation have not yet been explored, however, given its highly anionic charge, its stability in a calcium-nanoparticle form (62), and its wide expression profile, it is likely that polyP has multiple effects on this innate immune pathway, analogous to its role in coagulation.

## Relevance of polyP in Complement

In the face of its pro-coagulant and pro-inflammatory properties, what might be the physiologic relevance of polyP in complement activation? The complement-dampening effect of polyP does not entirely conflict with the defined role of polyP in other biological systems. PolyP is prominently expressed in several cellular compartments of prokaryotes, where it exhibits pro-survival properties as an energy source, a metal ion chelator, a molecular chaperone, in enhancing pathogenicity (70–72), and in some cases, protecting against complement mediated death (73).

One can speculate on how polyP and C1-INH might co-operate in host protection. C1-INH is synthesized by and found on the surface of endothelial cells (74). Endothelial cells also display abundant glycosaminoglycans on their surface as heparan sulfate. PolyP, released by activated cells and found at low concentrations in the blood of healthy individuals (75, 76) would be available to bind to C1-INH on the endothelium, where it could potentiate the function of C1-INH, allowing the C1-INH:polyanion complex to recruit and neutralize target proteases, such as C1s and/or MASP2. Binding of the polyanion first to C1-INH is required for optimal neutralization of C1s. Such an order of events would best keep complement activation in check. PolyP would also be positioned to dampen generation of MAC on the host cell surface. In such a scenario, the activated endothelial cell would be protected against host-mediated destruction, while retaining its prothrombotic and pro-inflammatory properties.

This model may also apply to other cells. As mentioned, polyP is abundant in platelets and released upon activation (54, 55, 77). C1-INH is also found in platelets, secreted and deposited on the activated platelet membrane (78). Although initially housed in separate granules, platelet activation results in colocalization of polyP and C1-INH in and on the platelet (66). High levels of polyP on activated platelets would therefore readily dampen complement activation by potentiating the inhibitory properties of C1-INH and interfering with the terminal pathway (69), overall protecting the underlying host cells from innate destruction, while allowing the platelets to promote hemostasis-thrombosis and inflammation. Interestingly, polyP also binds to FH (69) and may similar to C1-INH, coat and protect host cells (34) from complement activation, convertase assembly and MAC binding/integration. Disturbances in the release of adequate polyP might therefore be predicted to result in disease. This is in fact evident in patients with dense granule storage pool diseases (79) who have low platelet polyP (80) and exhibit a bleeding diathesis and organ dysfunction secondary to excessive inflammation.

### Taking Advantage of the Complement-Dampening Properties of polyP for Therapeutic Purposes

Immediate clinical application of the finding that polyP, a naturally occurring and easily synthesized polyanion that suppresses complement activation, is enticing but not without challenges. Depending on the length, dose and formulation, systemic delivery of polyP may entail risk of thrombosis. However, polyP has been administered safely *in vivo*, providing protection against endotoxin-induced sepsis in a mouse model (81). If validated, one could envisage using polyP for a wide range of disorders with excess complement activation. Our group has limited *in vivo* testing of polyP to study its role in protecting against age-related macular degeneration (AMD). AMD is a common cause of blindness where over-activation of complement is a major pathogenic driver (82). In a mouse model of laser-induced AMD, intravitreal administration of polyP dampened the pathologic neovascularization and complement deposition to a similar extent as currently used anti-VEGF targeted therapies (83). No adverse effects of polyP were observed, providing strong rationale for further exploration.

## Conclusion

In the last 20–30 years, major inroads have been made in delineating the molecular mechanisms by which complement, coagulation and inflammation intersect. The preceding discussion underlines the unique role that polyP plays in suppressing complement, while promoting coagulation and inflammation. Further understanding of how polyP modulates complement activation through the induction of structural changes in key factors in these different proteolytic cascades, and/or interactions with other proteins and cells, will reveal novel sites for therapeutic intervention for a range of thrombotic and inflammatory disorders.

## Author Contributions

The author confirms being the sole contributor of this work and has approved it for publication.

### Conflict of Interest Statement

The author declares that the research was conducted in the absence of any commercial or financial relationships that could be construed as a potential conflict of interest.
